# A novel sandwich-type terahertz metasurface sensor integrated ultrathin microfluidic channel for direct detection of aqueous solutions

**DOI:** 10.1515/nanoph-2025-0099

**Published:** 2025-06-06

**Authors:** Yunhao Cao, Hongshun Sun, Xubo Song, Zhihong Feng, Shixiong Liang, Yusa Chen, Lijun Ma, Liye Li, Wengang Wu

**Affiliations:** National Key Laboratory of Advanced Micro and Nano Manufacture Technology, School of Integrated Circuits, 12465 Peking University, 100871, Beijing, P.R. China; National Key Laboratory of Solid-State Microwave Devices and Circuits, Hebei Semiconductor Research Institute, 050051, Shijiazhuang, P.R. China; School of Microelectronics, Tianjin University, 300072, Tianjin, P.R. China; Beijing Advanced Innovation Center for Integrated Circuits, Beijing, P.R. China; Frontiers Science Center for Nano-Optoelectronics, 12465 Peking University, Beijing, P.R. China

**Keywords:** terahertz metasurface, microfluidic sensor, aqueous solution detection, high sensitivity, high quality factor

## Abstract

A considerable part of the energy of the traditional terahertz metasurface sensor located in the substrate/dielectric layer, which cannot contact and interact with the analyte, resulting in energy loss and greatly limiting the sensitivity of the sensor. In addition, the strong absorption of THz wave by water makes it difficult for THz metasurface sensor to achieve the direct detection of aqueous solution. In this paper, a novel sandwich-type THz metasurface microfluidic sensor with high sensitivity for direct detection of aqueous solution is proposed and verified. Numerical simulation results show the proposed sensor has a perfect absorption peak with high *Q*-factor of 67.55 and high *S* of 0.534 THz/RIU at 1.85 THz. The proposed sensor not only can confine all the energy in microfluidic channel greatly improving the energy utilization and light–matter interaction, but also precisely controls the liquid thickness at 3.1 μm greatly reducing the absorption of THz wave, which provides the foundation for realizing the direct detection of aqueous solutions with high sensitivity. Na^+^ aqueous solutions of different concentrations are selected as analytes to verify the performance and feasibility of the proposed novel sensor. The offset of Na^+^ aqueous solution with concentrations of 50, 100, 300, 500, 1,000 and 1,500 mmol/L is 13, 22, 46, 60, 93 and 115 GHz, respectively (using pure water as reference). The experiment and simulation of the sensor are basically consistent, which fully verifies the high sensing performance of the designed device and its applicability to the direct sensing of aqueous solution.

## 1 Introduction

The terahertz (THz) wave with a frequency of 0.1 THz–10 THz and a wavelength of 3 mm–0.03 mm is an electromagnetic wave between the microwave band and the infrared band, and thus has both electronic and optical characteristics [1]. The unique properties of THz wave, such as low photon energy, strong penetration and fingerprint spectrum of biological macromolecules, make it have great application potential in the field of sensing [2]. However, the interaction of free space THz wave with the analyte is too weak, which also leads to the problem of low spectral signal-to-noise ratio (SNR) and low sensitivity [3]. Electromagnetic metasurface is an artificially constructed material composed of periodic arrangements of subwavelength size meta-atoms [4], [5]. Metasurface can manipulate electromagnetic waves by controlling its meta-atom structure and its array arrangement, including arbitrarily controlling and manipulating the amplitude, phase and polarization of electromagnetic waves [6], [7], [8], [9], [10]. In addition, the metasurface also has the effect of local field enhancement, which greatly enhances the terahertz wave-matter interaction, and solves the limitations of free-space terahertz spectroscopy in the field of biochemical sensing to a certain extent [11].

In recent years, the combination of metasurface and terahertz spectroscopy technology has been applied in a series of sensing fields, and the analytes are mainly divided into several categories, including nano-scale biomolecules [12], [13], micron-scale cells and bacteria [14], [15], organic solvents [16], [17] and ionic solutions [11]. In 2021, Wang R.D et al. designed a transmission THz metasurface sensor based on the Q-BIC, with a theoretical sensitivity of 0.165 THz, which realized trace detection of interleukin-6 with a detection limit of 1 nM [7]. In 2023, Liu B.W. et al. designed an asymmetric ring-chain transmission THz metasurface sensor based on Q-BIC, with a theoretical sensitivity of 0.42 THz/RIU to achieve trace detection of cysteine. The lower limit detection (LoD) of Q-BIC resonance peak is 12.5 pmol/μL. Compared with the traditional dipole resonance peak, the lower detection limit is about 40 times higher [18]. In 2021, Zhang J et al. proposed a transmission terahertz metasurface sensor based on the EIT-like effect, with a theoretical sensitivity of 0.496 THz/RIU, for quantitative and qualitative detection and analysis of different glioma cells based on the change of two parameters including frequency and amplitude [15].

The sensors mentioned above are all for the detection and analysis of dry analytes. Most biological biomolecules and physiological environments are liquid environments, so it is necessary to conduct direct sensing of analytes in liquid environment. In recent years, many terahertz metasurface sensors with integrated microchannels have been reported. In 2016, S.J. Park et al. integrated a Polydimethylsiloxane (PDMS) microchannel with a height of 50 μm on an Inductor-Capacitor (LC) transmission THz metasurface to achieve the direct sensing detection of some common organic solvents [19]. To enhance the terahertz wave-matter interaction to achieve higher sensor sensitivity. In 2016, Hu X et al. for the first time replaced the dielectric layer of the traditional metal-media-metal (MIM) structure with a micron-scale microflow channel, and the measured sensitivity reached 0.22 THz/RIU [17].

In 2019, Lan F et al. improved the processing technology of the sensor mentioned in the Ref. [17] and proposed a dual-mode terahertz metasurface microfluidic sensor to detect and analyze bovine serum protein at different concentrations (the solvent is PBS buffer) [20]. Due to the strong absorption of terahertz waves by water, it is difficult to realize THz metasurface to directly detect aqueous solutions. At present, there are few implementations, and the available experiment data are very limited. Although the absorption of terahertz waves by water is strong, the sensing of aqueous solutions using terahertz metasurface is not completely unfeasible. It has been reported that spectral data can be obtained by controlling the optical path of terahertz waves in water within 50 μm [16], [19].

The reported data show that the refractive index of the aqueous solution is between 2.0 and 2.2 [19], [21], [22], [23], [24]. Therefore, there are two key points to realize the direct sensing of aqueous solutions with small dielectric environment changes in the terahertz band. One is that the smaller the optical path of the terahertz wave in water, the better, and the other is that the sensor must have a very high sensitivity to ensure that it is sensitive enough to changes in the tiny dielectric environment of aqueous solutions. However, sensitivity (*S*) and quality factor (*Q*-factor) are the key performance indexes for evaluating sensors, and the indexes of THz metasurface sensors mentioned above are not very high, so it is difficult to realize the direct sensing of aqueous solutions.

Based on the above analysis, to realize the direct sensing of aqueous solution by terahertz metasurface sensor, in this paper, a highly sensitive reflective terahertz metasurface microfluidic sensor for the direct sensing of aqueous solution detection is proposed and experimentally verified based on ultra-thin quartz. The sensor is formed by bonding an ultra-thin quartz based metasurface layer with a silicon pedestal layer etched by Micro-Electro-Mechanical Systems (MEMS) technology. The metal microarray layer and the metal reflector form a microflow channel region with a height of 3.1 μm. The strong field energy of the sensor is mainly concentrated in the microflow channel, which greatly enhances the terahertz wave-matter interaction. In order to verify the design and fabrication of the sensors, NaCl solutions of different concentrations are analyzed. The experimental results are basically consistent with the simulation results, which not only fully verifies the theoretical design and machining of the proposed sensor, but also verifies the high performance of the designed sensor and its applicability to aqueous solution sensing. Compared with the terahertz metasurface sensors reported in recent years, the sensitivity, quality factor and the figure of merit (*FOM*) of the sensor proposed in this paper have good performance, and have great application potential in the future of aqueous solution detection in the terahertz band.

## 2 Design and mechanism

### 2.1 Sensor design

**Figure 1:**
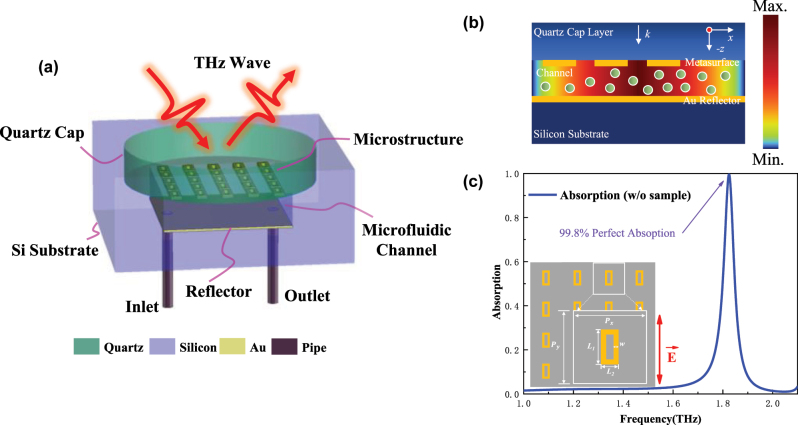
Schematic illustration and electromagnetic characteristics of the proposed sandwich-type THz metasurface microfluidic sensor. (a) Overall diagram (b) diagram of field energy distribution (c) unit structure and perfect absorption peak of the proposed sandwich-type THz metasurface microfluidic sensor.

The proposed sandwich-type THz metasurface microfluidic sensor consists of an ultra-thin quartz metasurface on the top layer, a silicon pedestal with steps and through-holes formed by etching three times by MEMS technology, and external pipes, as shown in Figure 1(a). The proposed sensor, essentially a THz metasurface perfect absorber (MPA), consists of a three-layer structure. The top layer is an ultrathin THz metasurface. The middle layer is a 3.1 μm microfluidic channel formed by etching a silicon substrate. The bottom layer is a gold reflective layer and a silicon substrate. Among them, the top metasurface substrate is selected as an ultra-thin quartz substrate with a dielectric constant of 3.75 + 0.004*i* and a thickness of 30 μm. The metal reflection layer and the metal microstructure layer are selected as the gold for its high stability and biocompatibility, the conductivity of gold is *σ* = 4.56 × 10^7^ S/m, and the thickness is 200 nm. The top metasurface layer based on ultra-thin quartz substrate (30 μm) is so fragile that the metasurface layer and silicon pedestal cannot be bonded by the traditional pressurized bonding method. The step design of the silicon pedestal provides condition for the bonding between the ultra-thin quartz metasurface layer and the silicon pedestal. Meanwhile, an ultra-thin microfluidic channel located in the strong field energy region is formed between the metal reflector and the metasurface, as shown in the field energy distribution diagram of the proposed sensor in Figure 1(b).

Like the traditional reflective-type metal–insulator–metal (MIM) metasurface, the proposed metasurface sensor mainly confines the energy between the metal microstructure and the metal reflector. However, the surface sample on the traditional MIM metasurface sensor cannot enter the dielectric layer and interact with the energy confined by the dielectric layer, which limits the sensitivity of the sensor. Unlike the traditional MIM metasurface sensor, the proposed sandwich-type THz metasurface microfluidic sensor replaces the dielectric layer of the traditional MIM metasurface structure with a microchannel, which can not only easily and accurately control the liquid thickness in the micron level reducing the absorption of terahertz waves by water and thus improving the signal-to-noise ratio of the experiment spectrum, but also the sample to be analyzed can be placed in the strong field energy area of the sensor to effectively improve the light–matter interaction, so as to achieve better detection effect and sensing performance under the condition of a small amount of the sample to be analyzed.

The THz metasurface element structure proposed in this paper is a hollow square ring structure, as shown in Figure 1(c). The structure parameters of the hollow square ring are as follows: horizontal/vertical element period *P*_*x*_ = *P*_*y*_ = 100 μm, the long side of the square ring *L*_1_ = 48 μm, the wide side of the square ring *L*_2_ = 24 μm, the line width of the square ring *w* = 7 μm. When the proposed reflective THz metasurface sensor is excited by THz wave the electric field direction is along the long side of the square ring, as shown by the red arrow in Figure 1(c), the design of the microfluidic channel with a height of about 3 μm realizes the impedance matching between the THz metasurface and the free space, thus resulting a perfect absorption, as shown in the blue simulation absorption spectrum in Figure 1(c).

Human physiological environment is mainly aqueous environment, and almost all small molecules that determine human physiological indicators are in aqueous environment, so the direct detection research of small molecule in water environment is very necessary. At present, THz metasurface sensors are mainly divided into two types, one is the traditional transmission-type metasurface sensor composed of common substrate such as quartz and metal microstructure, and the other is the traditional MIM reflective metasurface sensor with three layers of metal reflector layer, insulator layer and metal microstructure layer. If this two conventional terahertz metasurface sensors mentioned above want to analyze aqueous solutions directly, they must additionally integrate the microfluidic channel formed by the PDMS reverse mold, which controls the thickness of the water film to at least tens of μm. The limitation of THz wave–matter interaction in the sensor structure and the strong absorption of THz wave by water result in low sensitivity and poor signal-to-noise ratio of the signal.

**Figure 2:**
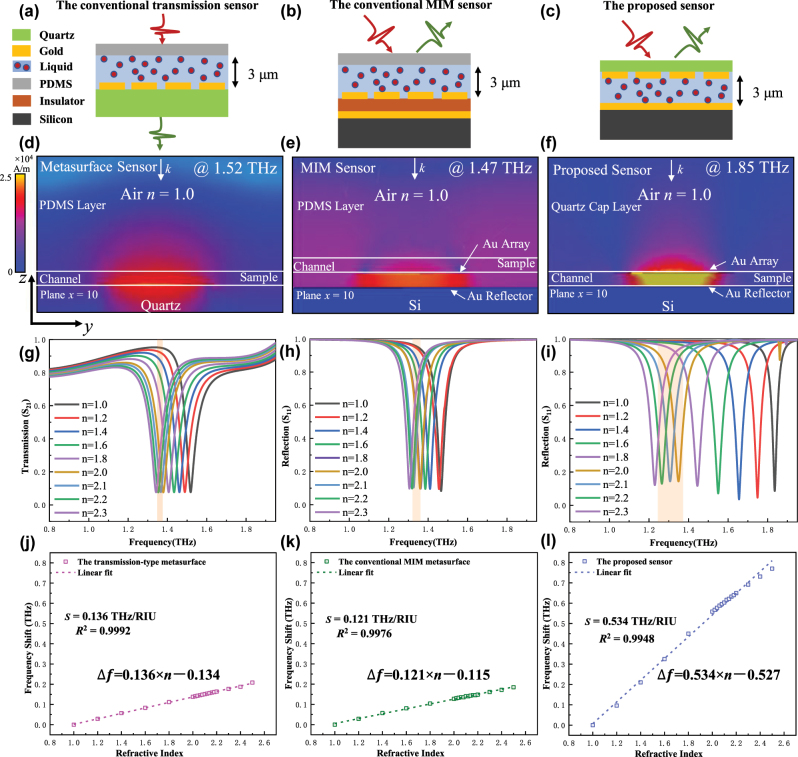
Under the conditions of the same THz element structure and the same liquids film. (a) Traditional transmission-type THz metasurface microfluidic sensor (b) traditional reflective-type MIM THz metasurface microfluidic sensor compared with (c) the proposed sandwich-type THz metasurface microfluidic sensor; (d) magnetic field distribution (g) simulated transmission *S*_21_ spectrum and (j) sensitivity fitting curve of the traditional transmission-type THz metasurface microfluidic sensor; (e) magnetic field distribution (h) simulated reflection *S*_11_ spectrum and (k) sensitivity fitting curve of the traditional reflective-type MIM THz metasurface microfluidic sensor; (f) magnetic field distribution (i) simulated reflection *S*_11_ spectrum and (l) sensitivity fitting curve of the proposed sandwich-type THz metasurface microfluidic sensor.

To better illustrate and verify the high sensitivity of the proposed sensor and its ability to make the direct detection of aqueous solution, the traditional transmission-type, traditional reflective and the proposed sandwich-type sensor were numerically calculated in the commercial electromagnetic simulation software CST 2022. In order to compare the performance differences between different sensor structures fairly and objectively, all the numerical calculations in Figure 2 are based on the square ring array in Figure 1(c) and the liquid film with the same thickness, besides which the thickness of PDMS microchannel is set to be the same as the thickness of the quartz substrate. Figure 2(a)–(c) shows the schematic diagrams of the traditional transmission-type THz metasurface microfluidic sensor, the traditional reflective THz metasurface microfluidic sensor and the reflective sandwich-type THz metasurface microfluidic sensor proposed in this paper, respectively.

As can be seen from the structure diagram of the three kinds different sensors in Figure 2(d)–(f), the strong field energy of the transmission-type metasurface is mainly distributed in the surface layer of the metal array and the substrate, and the strong field energy of the MIM reflective metasurface is mainly distributed in the surface layer of the metal array and the dielectric layer (mainly in the dielectric layer). The liquid sample loaded on the surface of the two kinds traditional sensors cannot enter the substrate or dielectric layer, which means that the liquids to be analyzed cannot fully interact with the strong field. In contrast, the sandwich-type sensor proposed in this paper not only can confine the energy mainly in the microchannel, but also has a higher field strength, which provides sufficient conditions for the full interaction of the liquids with the strong field explaining that the proposed sensor in this paper is 4 times more sensitive than the conventional transmission/reflective metasurface sensor structure, as Figure 2(h)–(l) shows.

Some published literature shows that the refractive index of water at 2 THz is about 2.0, while the refractive index of salt solutions with human physiological index concentrations is distributed between 2.0 and 2.2, as shown in the light orange region in Figure 2(h)–(j).Considering the resolution of the terahertz time domain spectroscopy system (THz-TDS) at the present stage, high sensitivity is necessary to achieve the direct detection of aqueous solution with a tiny refractive index changes (Δ*n* < 0.2). As shown in the light orange region in Figure 2(h) and (i), the testable spectral width left by the traditional transmission-type THz metasurface structure and the traditional MIM reflective metasurface structure for THz-TDS is too narrow, and the current THz-TDS cannot achieve such a high spectral resolution. As shown in Figure 2(j), the spectrum width of the calibration aqueous solution of the proposed sensor in the light orange region is about 150 GHz, which fully proves the proposed novel THz microfluidic sensor provides conditions for the direct detection of solutions with small dielectric difference. Figure 2 demonstrates the feasibility of direct aqueous solution detection only through frequency shift analysis, a detailed discussion of THz wave absorption by aqueous solutions can be found in Supplementary Material S4.

### 2.2 Mechanism

The proposed novel sandwich-type metasurface sensor is essentially a THz perfect absorber, and the absorption rate *A*(*ω*) of THz metasurface absorber can be calculated by the formula *A*(*ω*) = 1 − |*S*_11_(*ω*)|^2^ − |*S*_21_(*ω*)|^2^, in which *S*_11_(*ω*) and *S*_21_(*ω*) are reflection coefficient and transmission coefficient, respectively. The thickness of the gold reflector layer (200 μm) of the proposed sensor is much larger than the skin depth (<100 μm) of the THz wave at its operating frequency, so the transmission coefficient *S*_21_(*ω*) can be ignored, thus the absorption rate calculation formula can be simplified as *A*(*ω*) = 1 − |*S*_11_(*ω*)|^2^. And the simplified absorption formula shows that the reflection coefficient should be reduced to the maximum extent to achieve a perfect absorption so that as much as possible energy can be confined in the microfluidic channel. The key to achieve low reflection coefficient is make the relative impedance of the part of the sensor above the gold reflector including metasurface and microchannel match with that of the free-space.

Based on the effective medium theory, the relative impedance *Z*(*ω*) and the absorption coefficient *A*(*ω*) of the proposed sensor can be calculated based on the equations as follows:

(1) $$Z\left(\omega \right)=\sqrt{\frac{{\left(1+S_{11}\left(\omega \right)\right)}^{2}-S_{21}^{2}}{{\left(1-S_{11}\left(\omega \right)\right)}^{2}-S_{21}^{2}}}\approx \frac{1+S_{11}\left(\omega \right)}{1-S_{11}\left(\omega \right)}$$

(2) $$A\left(\omega \right)=1-{\left|S_{11}\left(\omega \right)\right|}^{2}=1-\frac{Z-1}{Z+1}$$

Based on the impedance of the free space (*Z*_0_ = 377 Ω is normalized as 1) and the numerically calculated *S*_11_(*ω*) of the proposed sensor, the relative impedance of the sensor at resonant frequency is calculated as 1.15 at the resonant frequency, approximately equal to 1.0, which means a very low reflectivity (*S*_11_(*ω*)).

**Figure 3:**
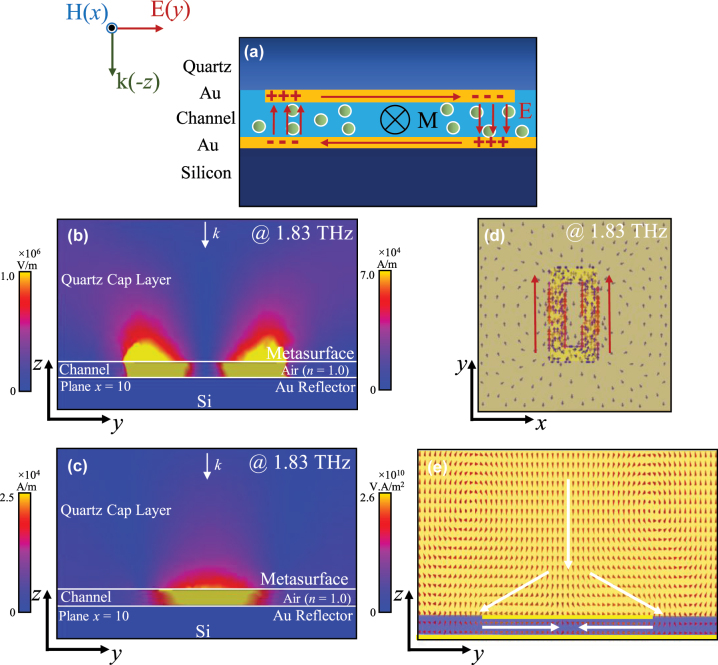
Field distributions and resonant mechanisms of the proposed sensor. (a) Artistic illustration of the electric and the magnetic field across the proposed sensor cross section with molecules from the channel representing the analyte. (b) Electric field distribution (c) magnetic field distribution (d) surface current distribution (e) power flow distribution of the proposed sensor at the resonant frequency.

When the proposed sensor is excited by terahertz waves with the E-Field parallel to the long side of the square ring (*Y*-axis), the edge field and resonant magnetic field above the proposed novel THz metasurface sensor are as Figure 3(a) shows. The electric field plays a key role in sensing analytes in the microfluidic channel. As shown in the Au microarray layer in the diagram of the *y*–*z* plane (plane *x* = 10) in Figure 3(a), the potential difference is formed on both sides of the long side of the square ring under the action of electric field, and the current along the *Y*-axis is formed at the same time. The metasurface element structure proposed in this paper is a symmetrical square ring, so two symmetrical current sections in the same direction will be formed on the two long sides of the square ring which generate a common dipole resonance mode, as shown in Figure 3(d). As shown in the diagram of the *y*–*z* plane (plane *x* = 10) in Figure 3(a), the potential difference between the two sides of the metal reflector is opposite to that of the upper metal microarray layer, and the current parallel to the *Y*-axis is opposite to the current in the microarray metal layer. The upper and lower current sections in opposite directions form a ring current, which induces a magnetic dipole at its center, which means a strong H-field area locals between the metesurface and the reflector which happens to be the microchannel area. According to the ampere rule, the magnetic field direction is −*x* direction.

Figure 3(b) and (c) respectively show the E-field distribution and H-field distribution of the proposed sensor (without sample) at the resonant frequency (@1.85 THz), which illustrates that strong E-field of the proposed sensor is mainly distributed in the area between metasurface and gold reflector (inside the microfluidic channel) and the surface layer of the metal microarray layer.

The strong H-field is mainly distributed in the region between the two layers of metal (inside the microfluidic channel), which is also very consistent with the above mechanism analysis that the loop current in the two layers of metal induces the magnetic dipole between metasurface and gold reflector. In general, the strong E-fields and H-fields of the proposed novel THz metasurface microfluidic sensor based on THz metasurface perfect absorber are basically distributed in the microchannel region which provides conditions for the full interaction of the measured aqueous solution with THz wave, and lays the foundation for the high sensitivity of the sensor.

**Figure 4:**
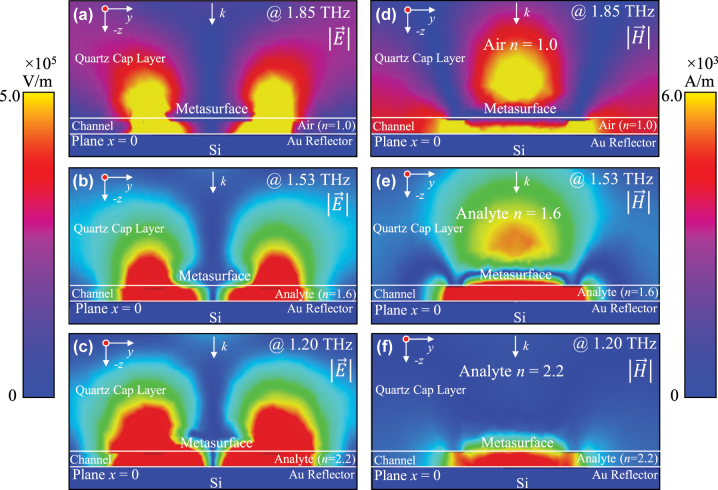
The corresponding E-field/H-field distribution of the proposed sensor with microfluidic channel filled with analytes of different refractive indices.

Figure 4 is the corresponding E-field/H-field distribution of the proposed sensor with microfluidic channel filled with analytes of different refractive indices. Figure 4(a)–(c) shows the E-field distribution of *y*–*z* plane (plane *x* = 0) respectively corresponding to analytes with different refractive indices *n* = 1.0 (@1.85 THz), *n* = 1.6 (@1.53 THz) and *n* = 2.2 (@1.20 THz) in the microfluidic channel, and Figure 4(d)–(f) shows the H-field distribution of *y*–*z* plane (plane *x* = 0) respectively corresponding to analytes with different refractive indices *n* = 1.0 (@1.85 THz), *n* = 1.6 (@1.53 THz) and *n* = 2.2 (@1.20 THz) in the microfluidic channel. The E-field resonance enhancement of the proposed sensor is due to the multi-path optical cycling in the microfluidic channel, which causes strong interaction between the analytes and the concentrated field in the microchannel and the edge field extending to the quartz cap layer of the sensor. Compared with the state of the sensor with air (*n* = 0), due to the presence of analytes in the microfluidic channel and the increase of refractive index (*n* = 1.6, *n* = 2.2), the overall strong field region is squeezed into a smaller spatial range in the *z* direction. As shown in Figure 4(d)–(f), the magnetic field distribution shows significant dependence on analyte refractive index. As refractive index of the sample *n* increases from 0 to 2.2, the H-field becomes increasingly confined within the microfluidic channel (Figure 4(f)), indicating improved energy utilization for detecting small dielectric variations in aqueous solutions.

As shown in Figure 2(i), when the refractive index of the liquid in the microflow channel gradually increases, the perfect absorption peak of the sensor will blue shift, based on which liquids can be analyzed. The sensing mechanism of the proposed sensor can be explained from two perspectives. First, different refractive index analytes will have different field distributions, and different electromagnetic field distributions correspond to different resonant frequencies, as shown in Figure 4. Second, the refractive index of the analyte directly affects the sensor’s resonance frequency. As the refractive index increases, the absorption peak shifts to lower frequencies (blue shift), enabling precise detection of liquid properties. In summary, the enhanced field in the microchannel and the edge field above the gold microarray play an important role in the sensing of the analyte layer illustrating the high sensitivity and sensitization mechanism of the proposed novel sandwich-type THz metasurface sensor which greatly improves the energy utilization rate and lays a foundation for high sensitivity detection of liquids with small dielectric difference, such as aqueous solution (*n* = 2.0 ∼ *n* = 2.2).

## 3 Fabrication and experiment

### 3.1 Sensor fabrication

**Figure 5:**
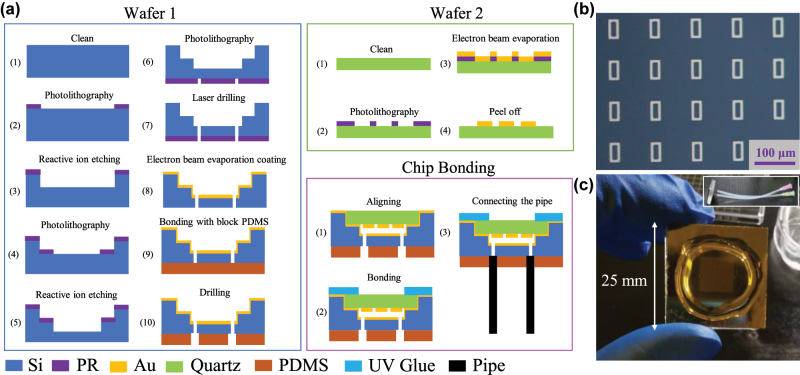
Fabrication process and optical characterization of the sensor. (a) Processing flow of the proposed sensor (b) optical micrograph of metasurface based on ultrathin quartz wafer (c) optical photograph of the proposed sensor.

As Figure 5(a) shows, the proposed sensor is fabricated using MEMS fabrication technology, the processing process can be mainly divided into three parts: the processing of silicon pedestal, the processing of metasurface based on ultrathin quartz wafer and the bonding between silicon pedestal and metasurface layer.

The processing of the silicon pedestal needs to be completed by three times of etching and one time metal deposition: (1) Cleaning the silicon wafer. (2–3) Spin coating photoresist, photoresist, graphic after development as the soft mask. The first Reactive ion etching (RIE) is carried out to form a cylindrical area with a diameter of 18 mm and a depth of 30 μm, which is slightly larger than the ultra-thin quartz wafer for embedding into the metasurface. (4–5) Secondary photolithography and Reactive iron etching (RIE) are performed to form a rectangular shape area of 10 mm × 10 mm × 3.3 μm, which is the microchannel area. (6–7) The third photolithography and etching are carried out on the other side of the silicon substrate to form through-holes for external microflow pipe to control the entry and exit of liquid. (8) Electron beam evaporation coating deposited 10 nm chromium and 200 nm gold on one side of the silicon step, forming the gold reflector in the MIM structure. (9–10) Bond blocks PDMS on the silicon substrate, and punch holes in the corresponding positions of the through silicon holes with the PDMS punch. The processing steps of the top ultra-thin quartz based metasurface are as follows: (1) Prepare the ultra-thin quartz wafer. (2) Spin coating photoresist, and photolithography, development, for the photoresist graphics. (3) Electron beam evaporation coating deposited 10 nm chromium and 200 nm gold successively. (4) Peel off excess photoresist to form a gold array pattern.

Considering the ultra-thin quartz base is too fragile, the traditional pressurized bonding method cannot realize the encapsulation between the silicon pedestal and the ultra-thin metasurface. In this paper, Ultraviolet (UV) shadowless adhesive is chosen to realize the bonding between silicon pedestal and the ultra-thin metasurface, meanwhile the problem of leakage is also solved, as shown in Figure 5(a). Figure 5(b) is the optical micrograph of the designed square ring array, and Figure 5(c) is the photograph of the sandwich-type THz metasurface microfluidic sensor proposed in this paper.

### 3.2 Experiment

**Figure 6:**
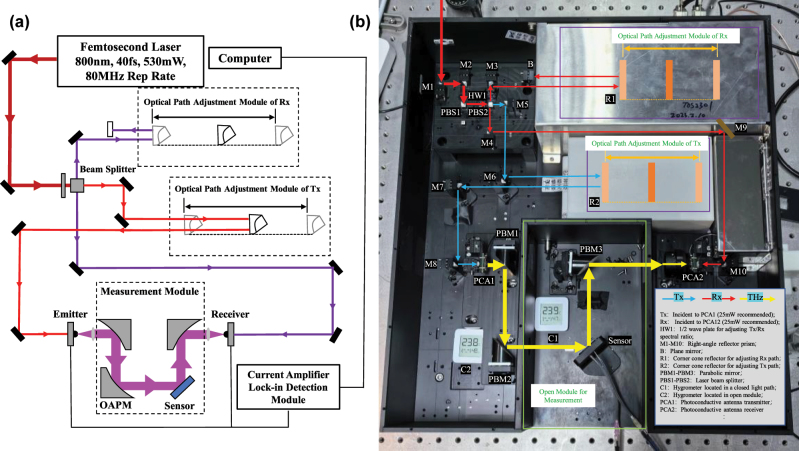
Experimental setup for reflective THz-TDS measurements. (a) Light path diagram and (b) optical photograph of the reflective THz time-domain spectroscopy system.

The measured system used in this paper is a reflective THz time-domain spectroscopy system (THZ-TDS) of BaTop THz Company in China. Figure 6(a) and (b) are the schematic diagram of the optical path and the optical photograph of THz-TDS system, respectively. The THz-TDS system supports three operation modes, including transmission mode, reflection mode (the incidence Angle of the reflection mode is fixed at 45°) and attenuated total reflection (ATR) mode. The resolution of this THz-TDS system can be adjusted up to 2.3 GHz, and the high signal-to-noise ratio can be maintained over a wide spectrum range of 0.1–3 THz. The terahertz beam is generated by the photoconductive antenna excited by the laser. And the terahertz beam spot diameter of the THz-TDS system is about 1 mm, which is much smaller than the 10 mm × 10 mm gold array area of the proposed sensor. Water vapor has a strong absorption characteristic of terahertz waves, and too much water vapor will result in too low signal-to-noise ratio of the test signal. To monitor the water vapor content in the optical path and test module in real time, two humidity/temperature sensors that can connect the phone via Bluetooth are placed in a closed optical path module and an open sensing test module, as Figure 6(b) shows. In order to completely ignore the effect of water vapor on the experiment results, a series of subsequent experiments were carried out at a temperature of 25 °C and a humidity of <5 %.

**Figure 7:**
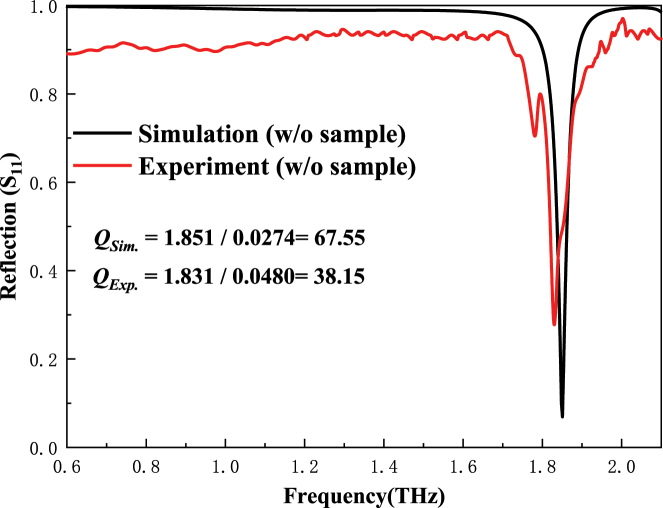
The measured *S*_11_ spectrum of the proposed sensor without sample compared with that of the simulation.

**Figure 8:**
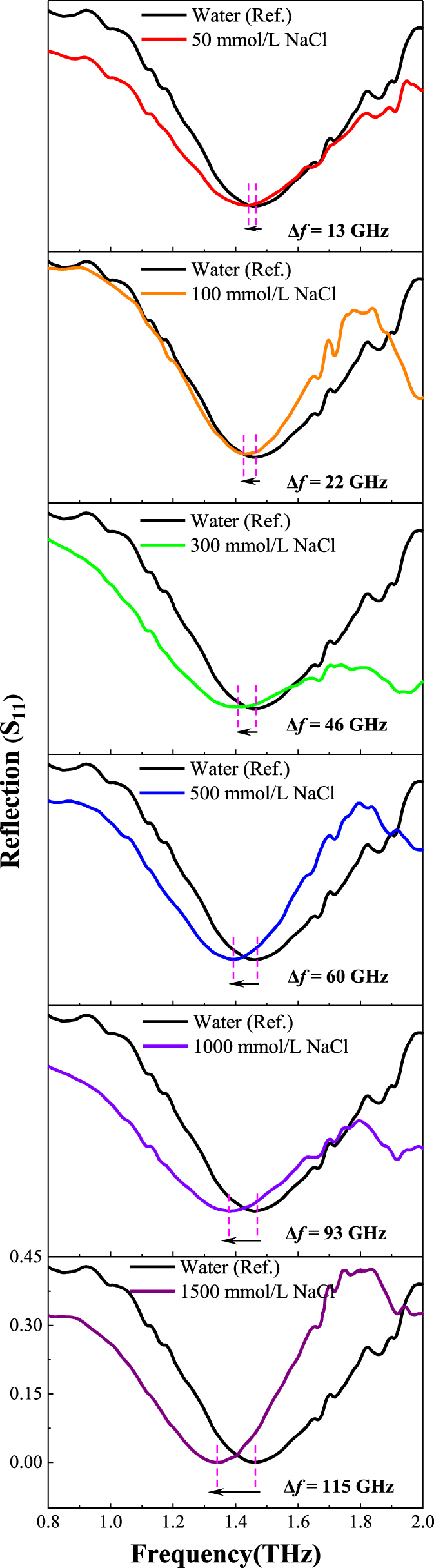
The measured *S*_11_ spectrum of the proposed sensor with the channel filled with 50 mmol/L, 100 mmol/L, 300 mmol/L, 500 mmol/L, 1,000 mmol/L and 1,500 mmol/L NaCl aqueous solution (pure water as the reference). Here, mmol/L is equivalent to mM (millimolar). Detailed specifications of concentration units are provided in Supplementary Material S2.

The comparison between the measured reflection spectrum and the simulated reflection spectrum of the proposed sensor (without analyte) is shown in Figure 7. The measured reflection spectrum of the sensor (without analyte) *f*_*Exp.*_ = 1.831 THz, which is slightly smaller than the simulated no-load resonance frequency *f*_*Sim.*_ = 1.851. The measured no-load quality factor *Q*_*Exp.*_ = 38.15 is smaller than the simulated no-load quality factor *Q*_*Sim*_ = 67.55. The experiment is basically consistent with the simulation. The slight difference between the simulation and experiment is mainly caused by fabrication errors and ambient noise. To verify the high performance of the proposed sensor and its sensing ability to make the direct detection of aqueous solution. A series of NaCl aqueous solutions of different concentrations is injected into the microfluidic channel to collect spectral data. The detailed sample preparation process can be seen in Supplementary Material S3 section. To better demonstrate the high sensitivity of the sensor to the change of analytes dielectric characteristics, the reflection spectra of NaCl solutions with different concentrations are compared with the reflection spectra of pure water, as shown in Figure 8.

**Figure 9:**
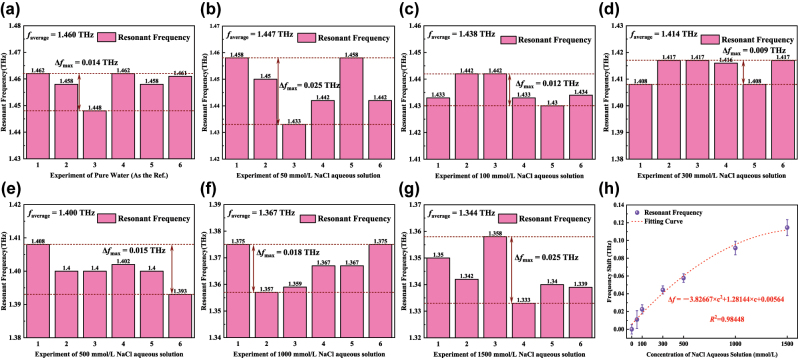
Experimental validation of aqueous solution sensing. (a)–(g) Measured resonant frequency of the proposed sensor with channel filled with different concentrations of NaCl aqueous solutions. (h) Frequency shift for different concentrations of NaCl aqueous solutions.

To ensure the reliability and repeatability of the experiment data, each concentration NaCl aqueous solution was measured at several times. The measured resonant frequencies in Figure 9(a)–(g) fully verifies the reliability and repeatability of the experiment. By calculating the average of the above experiment results, 50 mmol/L, 100 mmol/L, 300 mmol/L, 500 mmol/L, 1,000 mmol/L and 1,500 mmol/L NaCl aqueous solutions, compared with the reflection spectrum of pure water, have a red shift of 13 GHz, 22 GHz, 46 GHz, 60 GHz, 93 GHz and 115 GHz, respectively. And Figure 9(h) shows the shift trend of resonant peak conforms to the physical law which verifies the ability of the proposed sensor for the direct detection of aqueous solution with subtle change in dielectric environment. More importantly, the sensor can realize the *in situ* detection of 50 mmol/L Na^+^ lower than the normal physiological indexes of human serum, which is conducive to clinical screening of patients with hypernatronemia in the future, and has certain application significance for health monitoring.

Table 1 lists some representative THz metasurface microfluidic sensors reported in recent years, and compares them with the THz metasurface microfluidic sensors proposed in this paper. Table 1 shows that the three key performance indicators of the proposed sensor (*S*, *Q*-factor and *FOM*) have excellent performance.

**Table 1:** Comparison of several recently reported THz metasurface sensors.

Ref	Resonance type	Resonance position [THz]	Analyte	RI sensitivity [THz/RIU]	*Q-*factor	FOM
[25]	EIT-like resonance	1.02	Cancer cells (dry)	0.131	10 (Sim.)	1.09
					8.5 (Exp.)	
[26]	LSP resonance	1.55	/	0.066	32 (Sim.)	/
					None (Exp.)	
[2]	Guided mode resonance	0.6	SiO_2_/Ge thin film	0.06	10 (Sim.)	0.6
					6 (Exp.)	
[27]	Fano resonance-BIC	0.66	/	0.024	>75 (Sim.)	/
					6.45 (Exp.)	
[7]	TD resonance-BIC	1.17	Interleukin-6 (dry)	0.165	>100 (Sim.)	1.83
					6 (Exp.)	
[28]	Anapole resonance	0.26	/	/	>14.5 (Sim.)	/
					8.67 (Exp.)	
[29]	MPA	2.99	/	2.2	48.2 (Sim.)	/
					/(Exp.)	
[17]	MPA (with microchannel)	0.82	Organic solvent	0.22	>5 (Sim.)	0.66
					2.46 (Exp.)	
[30]	MPA (with microchannel)	0.81	/	0.16	28.2 (Sim.)	3.93
					19.8 (Exp.)	
[20]	MPA (with microchannel)	0.76	BSA solution	0.47	>5 (Sim.)	3.92
					6.33 (Exp.)	
[20]	MPA (with microchannel)	1.28	BSA solution	0.51	>10 (Sim.)	4.63
					11.64 (Exp.)	
This work	MPA (with microchannel)	1.83	Na^+^ aqueous solution	0.534	67.55 (Sim.)	11.13
					38.15 (Exp.)	

## 4 Conclusions

In this paper, a high sensing performance sandwich-type THz metasurface microfluidic sensor is proposed and fabricated, and it is proved that the proposed sensor can be used for the direct detection of aqueous solution by a series of experiments. The novel sensor is formed by bonding an ultra-thin quartz metasurface layer with a silicon pedestal layer etched three times by MEMS fabrication technology. The step design on the silicon base forms an ultra-thin microfluidic channel with a height of 3.1 μm between the metal microarray layer and the metal reflector layer, which effectively reduces the absorption of THz waves by the aqueous solution improving the signal to noise ratio. Compared with the traditional MIM/transmission metasurface sensor whose strong field energy is mainly concentrated in the dielectric layer that cannot be effectively utilized, the proposed sandwich-type THz metasurface microfluidic sensor basically confines the field energy in the microfluidic channel, which improves the energy utilization rate and greatly enhances the THz wave–matter interaction, laying a favorable foundation for the realization of high-performance sensing. The numerical calculation shows the proposed sensor has a perfect absorption peak with high *Q*-factor of 67.55 and high *S* of 0.534 THz/RIU at 1.85 THz, which means the proposed sensor has a very high figure of merit of 19.489. Compared with some recently reported THz metasurface microfluidic sensor, the three performance indexes of the proposed novel sensor all have better performance. To verify the excellent performance of the proposed sensor and its feasibility of detecting aqueous solutions directly, NaCl aqueous solutions with different concentrations are selected as analytes for experiment. Using pure water as the reference, NaCl aqueous solutions with 50 mmol/L, 100 mmol/L, 300 mmol/L, 500 mmol/L, 1,000 mmol/L and 1,500 mmol/L NaCl aqueous solutions have a red shift of 13 GHz, 22 GHz, 46 GHz, 60 GHz, 93 GHz and 115 GHz respectively, which conform to physical laws.

In summary, the experiment results of the proposed sensor are consistent with the simulation, which fully verifies the theoretical design and fabrication of the proposed sensor, which also verifies the high sensing performance of the proposed sensor and it can be used for the direct analysis of aqueous solution. In addition, the proposed sensor can realize the *in situ* detection of 50 mmol/L Na^+^ lower than the normal physiological indexes of human serum, which has great potential for clinical diagnosis of hypernatremia patients in the future.

## Supplementary Material

Supplementary Material Details
