# Supplementary material for: A novel sandwich-type terahertz metasurface sensor integrated ultrathin microfluidic channel for direct detection of aqueous solutions
Source: Nanophotonics. 2025 Jun 6;14(13):2331–43. doi: 10.1515/nanoph-2025-0099 (PMC12199555; doi:10.1515/nanoph-2025-0099)
Supplement: Supplementary file 1 — Supplementary Material Details [file j_nanoph-2025-0099_suppl_001.docx]

**Supplementary Material for**

A Novel Sandwich-Type Terahertz Metasurface Sensor Integrated Ultrathin Microfluidic Channel for Directly Detection of Aqueous Solutions

*Yunhao Cao^1^, Hongshun Sun^1^, Xubo Song^4^, Zhihong Feng^4^, Shixiong Liang^5^,*

*Yusa Chen^1^, Lijun Ma^1^, Liye Li^1^, Wengang Wu^1,2,3*^*

^1^ National Key Laboratory of Advanced Micro and Nano Manufacture Technology, School of Integrated Circuits, Peking University, Beijing 100871, P. R. CHINA

^2^Beijing Advanced Innovation Center for Integrated Circuits, P. R. China

^3^Frontiers Science Center for Nano-optoelectronics, Peking University, P. R. China

^4^National Key Laboratory of Solid-state Microwave Devices and Circuits, Hebei

Semiconductor Research Institute, Shijiazhuang, P. R. China, 050051

^5^School of Microelectronics, Tianjin University, Tianjin, P. R. China, 300072

* Corresponding author: [wuwg@pku.edu.cn](mailto:wuwg@pku.edu.cn)

**Part 1. List of Abbreviations**

Supplementary Table S1 shows the list of Abbreviations in the Manuscript.

**Supplementary Table S1: List of Abbreviations in the Manuscript**

| **Abbreviation** | **Full Term** | **Annotation** |
| --- | --- | --- |
| THz | Terahertz | "Terahertz (THz)" |
| SNR | Signal-to-Noise Ratio | "Signal-to-Noise Ratio (SNR)" |
| PDMS | Polydimethylsiloxane | "Polydimethylsiloxane (PDMS)" |
| MEMS | Micro-Electro-Mechanical Systems | "Micro-Electro-Mechanical Systems (MEMS)" |
| UV | Ultraviolet | "Ultraviolet (UV)" |
| RIU | Refractive Index Unit | "Refractive Index Unit (RIU)" |
| S | Sensitivity | "Sensitivity (S)" |
| Q-factor | Quality factor | " Quality factor (Q-factor)" |
| FOM | Figure of Merit | "Figure of Merit (FOM)" |
| THz-TDS | Terahertz Time-Domain Spectroscopy | "Terahertz Time-Domain Spectroscopy (THz-TDS)" |
| LC | Inductor-Capacitor | "Inductor-Capacitor (LC)" |
| MIM | Metal-Insulator-Metal | "Metal-Insulator-Metal (MIM)" |
| MPA | Metasurface perfect absorber | "Metasurface perfect absorber (MPA)" |

**Part 2. Description of NaCl concentration units**

In this study, all NaCl concentration units have been standardized to **mmol/L**, with the following equivalence:**1 mmol/L = 1 mM = 10⁻³ mM/mL = 10⁻³ mmol/mL**

**Rationale for Unit Selection**

Based on comprehensive literature review [1-4], mmol/L is the International System of Units convention for reporting physiological concentrations (e.g., saline solutions or electrolyte levels). This unit is:

- Widely adopted in clinical chemistry and biomedical research (e.g., serum Na⁺ levels are conventionally expressed in mmol/L).
- Compatible with journal-specific requirements for unit standardization.

**Part 3. Sample preparation process**

**Preparation of NaCl Aqueous Solutions (50 – 1500 mmol/L)**

**1. Materials**

- **Sodium chloride (NaCl)**: High-purity crystals (≥99.8%, CAS 7647-14-5)
- **Solvent**: Ultrapure water (resistivity ≥18.2 MΩ·cm, Milli-Q system)
- **Containers**: Sterile polypropylene centrifuge tubes (15 mL, Corning)
- **Equipment**:
  - Precision electronic balance (±0.1 mg)
  - Magnetic stirrer (with temperature control)
  - 0.22 μm nylon filters (sterile)

**2. Stock Solution (1500 mmol/L)**

1. **Weighing**:
   - Molar mass of NaCl = 58.44 g/mol
   - Mass required for 100 mL:

*m* =1.5 mol/L × 0.1 L×58.44 g/mol = 8.766 g

1. **Preparation**:
   - Dissolve 8.766 g NaCl in 80 mL ultrapure water with stirring (500 rpm, 25°C, 30 min).
   - Adjust to 100 mL final volume, then sterilize by 0.22 μm filtration.

**3. Gradient Dilution**

A **two-step dilution** protocol was adopted:

1. **Intermediate solution (150 mmol/L)**:
   - Mix 10 mL stock solution + 90 mL ultrapure water.
2. **Working solutions**:
   - Prepare target concentrations per Table 1:

| **Target [NaCl] (mmol/L)** | **Volume of 150 mmol/L (mL)** | **Volume of H₂O (mL)** | **Total Volume (mL)** |
| --- | --- | --- | --- |
| 50 | 3.33 | 6.67 | 10.00 |
| 100 | 6.67 | 3.33 | 10.00 |
| **Target [NaCl] (mmol/L)** | **Volume of 1500 mmol/L (mL)** | **Volume of H₂O (mL)** | **Total Volume (mL)** |
| 300 | 20.00 | 80.00 | 100.00 |
| 500 | 33.33 | 66.67 | 100.00 |
| 1000 | 66.67 | 33.33 | 100.00 |
| 1500 | 100.00 (undiluted stock) | — | — |

**4. Storage**

- **Short-term**: 4°C, protected from light (<1 week).
- **Long-term**: Aliquot and store at -20°C; thaw at 37°C before use (vortex for 10 s).

**5. Critical Notes**

- **Volume contraction**: For solutions ≥500 mmol/L, allow 30 min equilibration after dilution.
- **Crystallization risk**: Solutions >500 mmol/L may precipitate at 4°C; store at RT (≤25°C) and vortex before use.

**Part 4. Discussion on absorption of terahertz waves by water**

**（1） Theoretical Estimation**

In our microfluidic sensor design, the effective THz wave propagation pathlength (*d*) through water can be approximated as:

*d* = 2×3.1×$\sqrt{2}$ ≈ 8.76 μm ≈ 9 μm （rounded for calculation convenience）


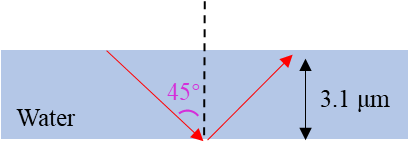


Figure 1. Diagram of terahertz wave propagation in water

Based on the Beer-Lambert Law ^[5-7]^, the absorption of a 9 μm water film at 1.83 THz is calculated as follows:

**1. Key Parameters**

- **Absorption coefficient (α)**：

326 cm⁻¹（measured value for water at 1.83 THz）^[7]^

- **Water film thickness (*d*)**：9 μm = 9 × 10⁻⁴ cm

**2. Calculation Procedure**

（1）**Transmittance (T)**：

*T* = *e^−αd^* = *e^−326×0.000 9^*= *e^−0.2934^* ≈ 0.746 (74.6%)

（2）**Absorption rate (A)**：

*A* = 1−*T* = 1−0.746 = 0.254 (25.4%)

**3. Key Conclusions**

- A 25.4% absorption rate indicates that:

(1) About 1/4 of incident THz wave energy is absorbed by the water film.

(2) About 74.6% signal intensity is retained, which remains suitable for most sensing applications.

(3) This provides the theoretical foundation for direct aqueous solution detection using our sensor design.

- **Comparison with other thickness:**

| **Water film thickness** | **Absorption rate**  **（1.83 THz）** | **Signal retention rate** |
| --- | --- | --- |
| 3 μm | 9.4% | 90.6% |
| **9 μm** | **25.4%** | **74.6%** |
| 50 μm | 80.4% | 19.6% |

**4. Experimental Validation**

- Our microfluidic channel height is 3.1 μm. After water injection:
  - Signal intensity decays from ~66% to 40 ± 5%
  - Measured signal retention: ~ 60 ± 7.5%
- This aligns well with the theoretical prediction (74.6% retention).
- The slightly lower experimental values are attributed to:
  1. Losses in the quartz cap layer
  2. Water vapor absorption in air (<5% humidity)
  3. Other system losses

**（2） Simulation Analysis**

In general, the real part of a sample’s complex refractive index primarily affects frequency shifts, while the imaginary part (extinction coefficient, κ) mainly influences amplitude changes. Therefore, in CST simulation software, the absorption of THz waves by water can be modeled by adjusting the material’s loss tangent (δ) to evaluate the effectiveness of the designed microfluidic device for aqueous solution testing. Figure 2 presents the simulated reflection spectra (*S_11_*) of the proposed THz metasurface microfluidic sensor with varying dielectric loss (δ) of the analyte. However, THz absorption in water is predominantly caused by hydrogen bonding, which cannot be precisely modeled in CST. Thus, this approach serves only as supplementary analysis and is less accurate than the theoretical calculations described above

Figure 2. The calculated reflective spectra (*S_11_)* of the proposed THz ESR-absorber biosensor with the variation of dielectric loss (*δ*) of the analyte.

**Summary Statement:**

In summary, the proposed terahertz metasurface sensor with an integrated ultra-thin microfluidic channel has demonstrated its capability for direct aqueous solution detection through comprehensive theoretical analysis, numerical simulations, and experimental validation. The design maintains sufficient signal integrity while achieving high sensitivity, thereby establishing a novel approach for terahertz-based analysis of aqueous solutions and high-water-content liquid samples in future applications.

**References**

1. H. J. Adrogué and N. E. Madias, "Hyponatremia," N. Engl. J. Med. 342(21), 1581-1589 (2000).
2. R. H. Sterns, "Disorders of plasma sodium—causes, consequences, and correction," N. Engl. J. Med. 372(1), 55-65 (2015).
3. M. Chavez, L. Chen, J. M. Gillet, et al., "Stable expression of large transgenes via the knock-in of an integrase-deficient lentivirus," Nat. Biomed. Eng. 7(5), 661-671 (2023).
4. H. Sun, Y. Cao, X. Song, et al., "A high-efficiency terahertz sensor based on surface lattice resonance metasurface for biochemical detection," Sens. Actuators A Phys. 377, 115711 (2024).
5. J. Xu, K. W. Plaxco, and S. J. Allen, "Absorption spectra of liquid water and aqueous buffers between 0.3 and 3.72 THz," J. Chem. Phys. 124(3), 036101 (2006).
6. J. Balakrishnan, B. M. Fischer, and D. Abbott, "Fixed dual-thickness terahertz liquid spectroscopy using a spinning sample technique," IEEE Photonics J. 1(2), 88-98 (2009).
7. S. J. Park, S. A. N. Yoon, and Y. H. Ahn, "Dielectric constant measurements of thin films and liquids using terahertz metamaterials," RSC Adv. 6(73), 69381-69386 (2016).
